# Trametinib-induced Hyponatremia in a Patient With Craniopharyngioma and Diabetes Insipidus

**DOI:** 10.1097/MPH.0000000000003116

**Published:** 2025-09-03

**Authors:** Buddhi Gunasekara, Harriet Gunn, Arif H.B. Jalal, Darren Hargrave, Hoong Wei Gan

**Affiliations:** * Department of Endocrinology, Great Ormond Street Hospital for Children NHS Foundation Trust; † Department of Endocrinology, University College London Hospitals NHS Foundation Trust; ‡ Genetics and Genomic Medicine Research and Teaching Department, University College London Great Ormond Street Institute of Child Health; ¶ Developmental Biology and Cancer Research and Teaching Department, University College London Great Ormond Street Institute of Child Health; § UCL Medical School, University College London; ∥ Department of Oncology, Great Ormond Street Hospital for Children NHS Foundation Trust, London, UK

**Keywords:** craniopharyngioma, trametinib, MEK inhibitor, diabetes insipidus, desmopressin

## Abstract

Adamantinomatous craniopharyngiomas (ACPs) are rare, sellar-suprasellar benign tumors that cause considerable morbidity and mortality due to local invasion and treatment-related damage to surrounding structures, including central diabetes insipidus (CDI). Trametinib is a highly selective inhibitor of MEK1 and MEK2, which has been evaluated in both adult and pediatric cancers/ tumors with activation of the oncogenic mitogen-activated protein kinase (MAPK) pathway. Despite being thought to have fewer side effects than conventional cytotoxic chemotherapy, off-target toxicities such as hyponatremia have been described. The use of MEK inhibitors in ACPs are limited to case reports and a phase II trial is currently underway. We report a pediatric patient with multiply progressive ACP and known brittle CDI who developed severe hyponatremia associated with a significant decrease in desmopressin dosing after starting trametinib and a rapid rebound of desmopressin requirement with its cessation. We recommend close monitoring of serum sodium levels and a review of desmopressin doses in patients with CDI when started on treatment with MEK inhibitors.

## BACKGROUND

Craniopharyngiomas are rare epithelial tumors of the sellar and parasellar region, histologically of low-grade (WHO grade 1, according to the latest WHO classifications of CNS tumors 2021).^1^ They constitute 5% to 10% of brain tumors in children. Although histologically benign, craniopharyngiomas cause considerable morbidity and mortality due to their location close to vital structures, resulting in tumor- and treatment- related damage from compression of the optic pathways, obstruction of the third ventricle, injury to the hypothalamo-pituitary axis leading to hypopituitarism, central diabetes insipidus (CDI) and hypothalamic dysfunction, including hypothalamic obesity. Main treatment modalities primarily include subtotal or complete resection of the tumor with or without radiation therapy. Conventional chemotherapy has no place.

Craniopharyngiomas are divided histologically into adamantinomatous (ACP) and papillary (PCP) subtypes. ACPs are more common in childhood and are predominantly cystic tumous caused by somatic mutations in *CTNNB1* (encoding β-catenin) resulting in activation of the Wnt pathway. PCPs mostly harbor somatic *BRAF* V600E mutations leading to activation of the mitogen-activated protein kinase (MAPK) signaling pathway.^2^ In one third of ACPs the *CTNNB1* mutation is not identified, suggesting other genetic/ epigenetic events might also cause *WNT* activation. Recently, involvement of the MAPK/ERK pathway in the tumorigenesis of ACP has been demonstrated, which potentially allows for novel therapeutic small molecules, such as the MEK inhibitor trametinib to be explored.^1,3^

Hyponatremia with trametinib monotherapy has been reported, particularly in the context of the treatment of low-grade gliomas.^4,5^ Hyponatremia can cause a wide spectrum of clinical symptoms, including nausea, headaches, asthenia, confusion, seizures, and even death. Here, we illustrate a case where the use of trametinib treatment for ACP resulted in significant changes in desmopressin requirement in a patient with brittle CDI.

## CASE REPORT

An 11-year-old girl was initially diagnosed with an ACP at age 3.5 years. She presented with a 6-month history of worsening daily headaches and longstanding vision loss more pronounced in her left eye. MRI brain showed a sellar and suprasellar predominantly cystic mass, consistent with craniopharyngioma. She underwent open decompression and open drainage of the cyst.

Optic atrophy, growth, and thyroid-stimulating hormone deficiencies were already present at diagnosis. Postoperatively, she was diagnosed with panhypopituitarism, including CDI. She commenced regular desmopressin 6 weeks following surgery, and subsequently developed hypothalamic obesity and impaired glucose tolerance.

Due to multiple disease progressions, she underwent several decompressive surgeries, proton beam therapy and Ommaya reservoir insertion at age 4 years. She developed a left hemispheric stroke post-irradiation at age 5.5 years, which required aspirin and external carotid—internal carotid bypass. Since age 9 years, ongoing multiple cystic and solid progressions required frequent decompressive surgeries. Post-surgery, she developed a right hemispheric stroke at age 11 years. Analysis of the resected tissue revealed a missense variant c.100G>A in the *CTNNB1* gene.

During her illness, she had multiple episodes of adrenal crises, and her CDI became increasingly difficult to manage, requiring gradual escalation in desmopressin doses up to 1400 micrograms per day in 5 divided doses. She also developed increasing lethargy and headaches, and required cyst aspiration every 7 to 10 days, providing temporary but not sustained improvement, significantly and negatively impacting her quality of life.

Due to ongoing difficulties in managing multiple tumor progressions and demonstration of positive immunohistochemistry for phospho-ERK, based on previous publications compassionate access to the MEK inhibitor, trametinib, was sought as it was thought this may be effective.^3,6,7^

At commencement, she had a serum sodium of 132 mmoL/L. After 1 week, she presented with abdominal pain, diarrhea, drowsiness, and collapse. Her serum sodium level was 120 mmoL/L on admission, whereby desmopressin was withheld.

Her desmopressin requirement was gradually restarted and she was stabilized on a total daily dose of 50 µg in 2 divided doses (3.6% of her original dose) to maintain eunatremia. She encountered well-reported side effects of trametinib, including paronychia, an erythematous rash (responding to low-dose topical hydrocortisone and regular emollients) and gastrointestinal side effects of acute abdominal cramps followed by loose stools managed by loperamide.

**FIGURE 1:**
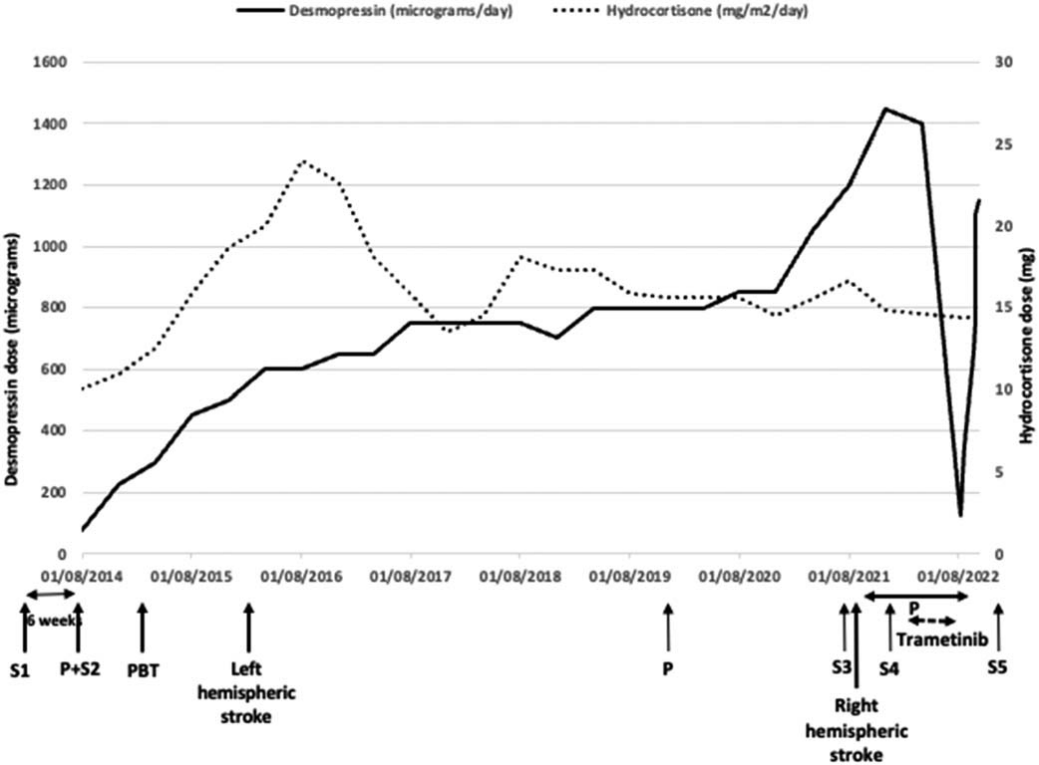
Desmopressin and hydrocortisone requirement with disease progression. P – progression of craniopharyngioma, PBT – proton beam therapy, S1 – open decompression and cyst fenestration, S2 – cyst fenestration, S3 – debulking, S4 – Ommaya reservoir insertion, S5 – subtotal resection. Following S4, the patient required multiple cyst aspirations through her Ommaya reservoir due to multiple cystic progressions.

Trametinib therapy was discontinued after 3 months due to treatment failure and ongoing side effects. This led to a rapid rebound of her desmopressin requirement up to 1150 µg per day in 5 divided doses (80% of her pre-Trametinib dose) (Fig. 1).

## DISCUSSION

The Ras-regulated RAF/MEK/ERK pathway is known to regulate key cellular functions, including proliferation, survival, differentiation, angiogenesis, and migration.^4^ Persistent hyperactivation of the MAPK pathway has been shown to be an oncogenic driver in several adult and pediatric cancers. This has led to the development and approval of several small molecular MAPK inhibitors. Trametinib is one of a group of highly selective inhibitors of the mitogen-activated protein kinase kinases 1 and 2 (MEK 1 and MEK2). Trametinib has been evaluated in pediatric clinical trials for tumors with activation of the MAPK pathway and has been recently authorized, in combination with the BRAF inhibitor dabrafenib, for the management of childhood gliomas harboring *BRAF* V600E mutations.

Common but usually low-grade and manageable toxicities of trametinib include acneiform eruptions, eczematous-like rash, paronychia, diarrhea, nausea and fatigue.^8^ Less common effects include ocular toxicity, cardiac toxicity, nephrotoxicity, weight gain and previous first reports of trametinib-associated hyponatremia.^4,9,10^

Of note, MEK inhibitors are not considered standard treatment for the treatment of ACPs which do not typically harbor BRAF V600E mutations unlike PCPs more commonly seen in adults.^11^ However, recent data suggests that ACPs do show evidence of MEK/ERK pathway activation, similar to the immunohistochemistry findings in our patient’s tumor tissue.^3,7^ Previous authors have shown that MEK inhibition may be effective in the management of ACPs^3,6,7^ and a phase II trial of the MEK inhibitor binimetinib is currently underway (NCT05286788). In our patient, a trial of trametinib was only considered on a compassionate basis due to the cumulative morbidity of multiple neurosurgical procedures, previous cerebrovascular disease post-irradiation, and brittle control of her hypothalamo-pituitary endocrine dysfunction.

Our patient presented with common manageable features of trametinib toxicity as well as acute severe hyponatremia. Before trametinib, she required high doses of desmopressin to maintain serum sodium within the normal range. Within a week of starting trametinib therapy, her desmopressin requirement went down to 3.6% of the original dose and with the cessation of therapy it rapidly rebounded.

Hyponatremia associated with trametinib monotherapy is potentially increased when combined with BRAF inhibitors.^12^ A study of endocrine effects of MEK and BRAF inhibitor therapy conducted in the same center to the present case, reported 16% (7/43) cases had hyponatremia during combined treatment with trametinib and a BRAF inhibitor (dabrafenib) and 13% (4/29) had hyponatremia with trametinib monotherapy.^13^ Three of these patients had CDI.

The exact mechanism by which MEK inhibitors cause hyponatremia remains unknown, although it is theorized that BRAF/MEK inhibitors lead to hyponatremia and water retention (dilutional hyponatremia) by activating aquaporin 2 (AQP2) trafficking from its intracellular compartment to the apical cell membrane of the renal tubule.^9,12^ In the renal collecting ducts, vasopressin regulates water permeability through AQP2. Some studies have shown that MAPK pathways may be involved in the osmoregulation of AQP1, which are in the proximal tubule, loop of Henle and expressed in the central nervous system (CNS), where it appears to be involved in cerebrospinal fluid secretion. Therefore, it is possible that MEK inhibitors disrupt the normal regulation of not just renal but also CNS AQP physiology.^9^ As a result, this could additionally result in fluid accumulation in the CNS.

Some observations suggest a link between osmotic dysregulation, altered renal sodium channel (ENaC) expression and ERK phosphorylation.^14^ The MAPK pathway regulates ENaC in the tubular collecting duct. BRAF and MEK downregulate ENaC activity and lower tubular sodium reabsorption. According to some hypotheses, BRAF/ MEK inhibitors activate ENaC and cause iso-osmolar sodium reabsorption, which also may lead to increased AQP2 trafficking to the cell membrane and enhanced water reabsorption. The overall effect is enhancing water reabsorption leading to dilutional hyponatremia. Studies recommend fluid restriction to treat trametinib-induced hyponatremia.^12^

In conclusion, selective MEK inhibitor therapy can cause hyponatremia due to fluid retention. Patients with known CDI need close sodium monitoring and close review of desmopressin doses when commencing trametinib under the supervision of a pediatric endocrinologist familiar with the management of hypothalamo-pituitary dysfunction in the context of suprasellar tumors.
